# Prophylactic monoclonal antibodies against respiratory syncytial virus in early life: An in‐depth review of mechanisms of action, failure factors, and future perspectives

**DOI:** 10.1111/pai.70257

**Published:** 2025-12-05

**Authors:** Etienne Bizot, Vincent Portet Sulla, Christelle Vauloup‐Fellous, Marie‐Anne Rameix‐Welti, Vincent Gajdos, Nabila Seddiki

**Affiliations:** ^1^ Inserm, CEA, Center for Immunology of Viral, Auto‐Immune, Hematological and Bacterial Diseases » (IMVA‐HB/IDMIT/UMRS1184) Université Paris‐Saclay Fontenay‐Aux‐Roses, Le Kremlin‐Bicêtre France; ^2^ General Pediatrics and Pediatric Emergencies, Antoine‐Béclère Hospital Paris Saclay University Hospital, AP‐HP Clamart France; ^3^ Division of Virology, WHO Rubella National Reference Laboratory, National Reference Laboratory for Perinatal Viral Infections, Department of Biology Genetics Paris Saclay University Hospital, AP‐HP Paris France; ^4^ Virology Department Hôpital Ambroise Paré, Paris Saclay University Hospital, AP‐HP Paris France; ^5^ Centre for Research in Epidemiology and Population Health, INSERM Villejuif France; ^6^ IHU‐SEPSIS Comprehensive Center Garches France; ^7^ Immunology and Pathogenesis Program Kirby Institute, UNSW Sydney Sydney New South Wales USA

**Keywords:** bronchiolitis, monoclonal antibody, pediatric, prevention, RSV, vaccine

## Abstract

The burden of respiratory syncytial virus (RSV) remains a major global health concern in early childhood, responsible for substantial morbidity, hospitalizations, and deaths, particularly in infants under 6 months. For over two decades, palivizumab was the only monoclonal antibody (mAb) available for prophylaxis, restricted to high‐risk groups due to cost and limited duration of protection. Recent advances in structural virology and antibody engineering have led to the emergence of long‐acting mAbs, notably nirsevimab and clesrovimab, which offer single‐dose seasonal protection and are now shifting RSV prevention strategies from high‐risk targeting to universal infant immunization. These antibodies act by locking the RSV fusion (F) protein in its pre‐fusion conformation, thereby preventing viral entry, with nirsevimab targeting site Ø and clesrovimab targeting site IV. Real‐world implementation has demonstrated remarkable reductions in RSV hospitalizations, aligning with clinical trial results, while large‐scale safety data support their favorable tolerance. However, the paradigm shift brings new challenges: understanding breakthrough infections, assessing long‐term immune imprinting, and anticipating viral evolution under immunological pressure. The risk of antigenic escape and the consequences of passive immunization on long‐term B‐cell memory development—especially in breakthrough cases—remain critical and underexplored immunological frontiers. This review explores the molecular and immunological underpinnings of RSV‐targeted mAbs, evaluates current real‐world evidence, and outlines future directions—including bispecific antibodies and nanobody‐based therapies—that could further transform RSV prophylaxis. Sustained genomic surveillance and a deeper understanding of host immunity will be crucial to preserve the long‐term efficacy of these innovations in pediatric infectious disease prevention.

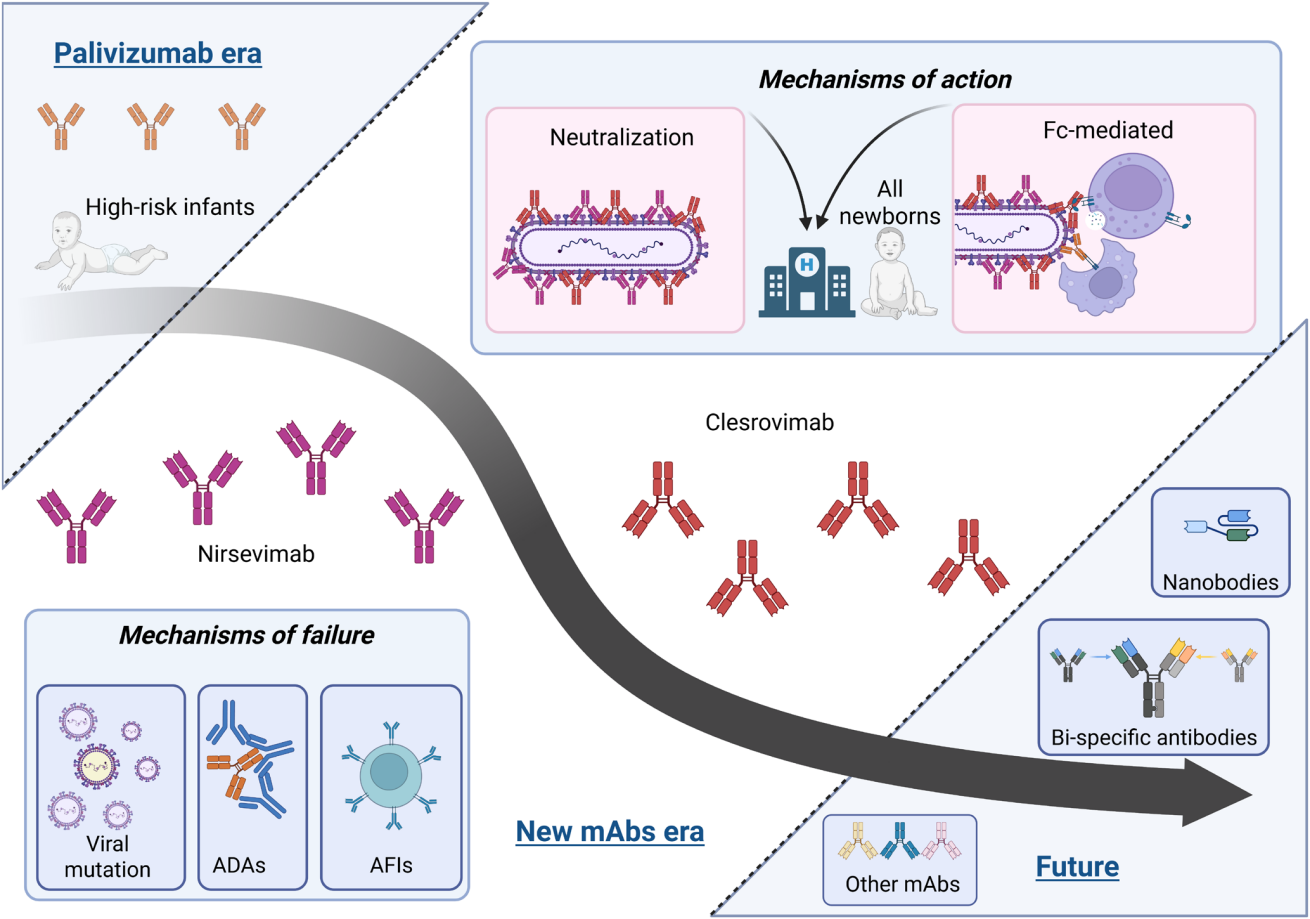

## Graphical abstract

Evolution of RSV immunoprophylaxis: from the palivizumab era to next‐generation monoclonal antibodies and future perspectives. Created in BioRender.

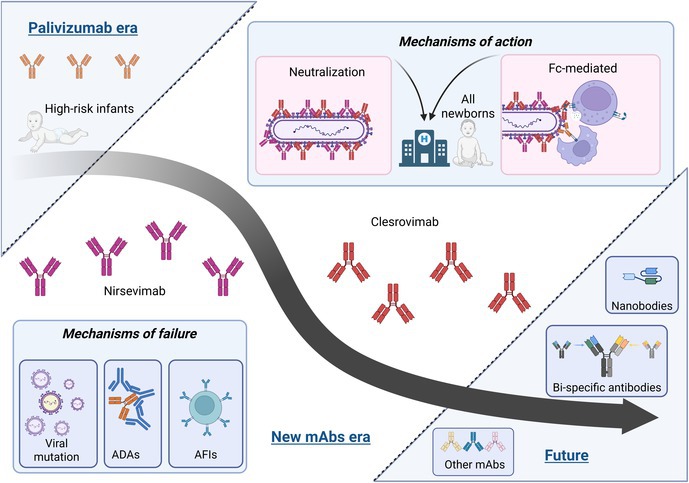

**Key message**

Long‐acting monoclonal antibodies have transformed RSV prevention, moving from high‐risk prophylaxis to universal infant protection. By clarifying their mechanisms of action, reasons for breakthrough infections, and long‐term immunological implications, this review provides a framework to safeguard their impact. It also highlights innovative strategies that will shape the future of pediatric infectious disease prevention.

## 1 INTRODUCTION: TRANSFORMING RSV PREVENTION: THE RISE OF PASSIVE IMMUNIZATION

**FIGURE 1:**
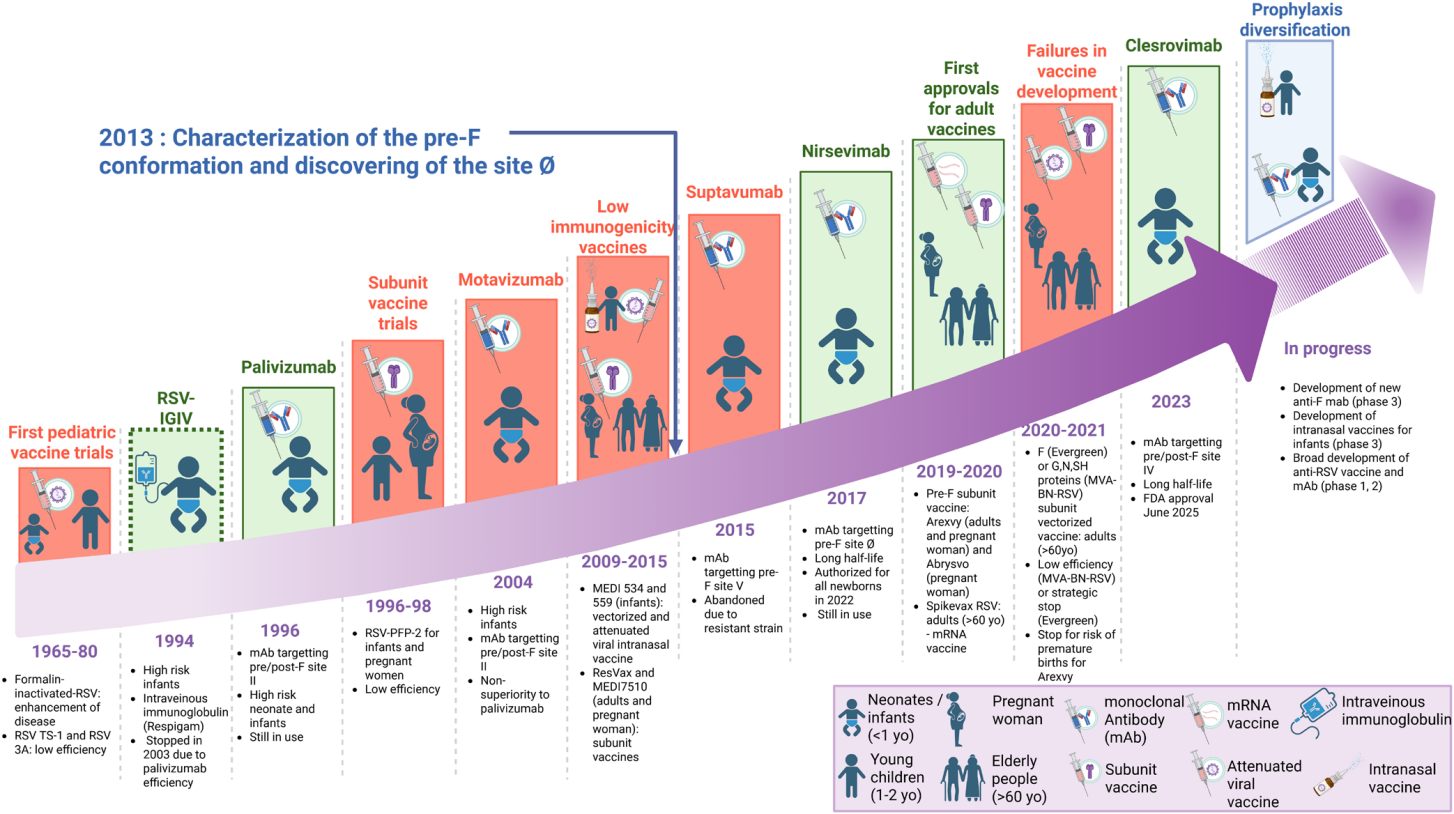
Road to successful immunoprophylaxis 1965–2025. The 1960s saw a tragic setback when a formalin‐inactivated RSV vaccine (1966) caused enhanced respiratory disease in infants (≈80% hospitalization rate, with two deaths).^9^ This failure halted early vaccine efforts and shifted focus to passive prophylaxis, culminating in the 1998 approval of palivizumab – a monoclonal antibody (mAb) to RSV F protein – for high‐risk infants.^13^ A breakthrough in 2013 revealed the pre‐fusion conformation of RSV F and a new neutralization‐sensitive epitope (“antigenic site Ø"), enabling structure‐based vaccine and mAb designs.^16^ Subsequent clinical trials delivered mixed results: for example, the investigational mAb suptavumab did not prevent RSV in phase 3 (ineffective against RSV‐B subtype due to dominance of resistant variants).^10^ By 2023, major advances reached fruition: the long‐acting mAb nirsevimab was approved to protect all infants through their first RSV season,^17^ and the first vaccines for older adults (pre‐fusion F protein–based subunit vaccines) achieved FDA licensure.^18^ Current preventive strategies are expanding to maternal immunization and novel platforms, including an mRNA‐based RSV vaccine (mRNA‐1345),^19^ as well as intranasal live‐attenuated vaccines for infants that are immunogenic but still under evaluation.^20^ Created in BioRender.

Respiratory syncytial virus (RSV) is the leading cause of acute lower respiratory tract infections (LRTI) in infants and young children worldwide. The epidemiological burden is immense: RSV is estimated to cause around 33 million LRTI cases, 3.6 million hospitalizations, and more than 100,000 deaths in children under five each year.^1^, ^2^ Primary infection, which consistently occurs before the age of two,^3^ is mostly benign, but can be particularly severe in infants under 6 months, of whom an estimated 1 in 28 deaths is attributable to RSV worldwide.^1^ Ninety‐seven percent of deaths occur in low‐ and middle‐income countries^1^ where access to supportive care (nutritional support, oxygen therapy, respiratory assistance), which constitutes the main care during infection, is limited.^4^ Furthermore, severe RSV infections in early life have been consistently associated with an increased risk of recurrent wheezing and later asthma, highlighting the long‐term respiratory consequences of early viral injury.^5^, ^6^ In parallel, RSV represents a major driver of inappropriate antibiotic use in infancy, with a substantial proportion of antibiotic prescriptions for bronchiolitis occurring in the absence of bacterial co‐infection further amplifying its public health impact.^7^ For decades, the development of vaccines or preventive monoclonal antibodies (mAbs) has been marked by insufficient results in terms of immunization,^8^ exacerbation of the disease^9^ or resistance acquired by RSV (Figure 1).^10^ Among immunoglobulin‐based therapies, only intravenous immunoglobulin (Respigam) has historically secured a role in the RSV preventive arsenal,^11^, ^12^ but it was rapidly replaced by palivizumab, a humanized monoclonal antibody (mAb) approved in 1998,^13^, ^14^ due to superior efficacy and specificity. Palivizumab use was limited to high‐risk infant populations (premature infants, children with chronic lung disease or congenital heart disease), due to its high cost and the requirement for monthly injections to maintain protection (Table 1). As a result, the vast majority of healthy, full‐term infants remained unprotected, despite being responsible for the majority of RSV‐related hospitalizations and infections.^15^

**TABLE 1:** Comparison of the three major commercialized anti‐RSV monoclonal antibodies.

	Palivizumab	Nirsevimab	Clesrovimab
Target epitope	F protein, Antigenic Site II	Pre‐fusion F protein, Site Ø	F protein, Antigenic Site IV
Primary in vivo mechanism	Neutralization; likely enhanced by Fc‐functions	Neutralization‐Dominant (Fc‐functions not required for optimal protection)	Presumed Neutralization; role of Fc‐functions not fully elucidated
Relative neutralization potency	Baseline	High (50–100× > Palivizumab)	High (16–23× > Palivizumab)
Key in vitro Fc functions	ADCC, ADCP, ADCD, Complement Activation	ADCP, ADCD (ADCC lower than Palivizumab)	Presumed
FcγR binding profile	Binds activating/inhibitory FcγRs	Binds FcγRs; modest reduction vs. precursor due to YTE	Not known
Half‐life	~20 days	~71 days	~45 days
Basis for half‐life	Native IgG1	YTE Modification in Fc Region	YTE Modification in Fc Region
Approved patient population	High‐risk infants	All infants; select children in 2nd season	Investigational; proposed for all infants in 1st season
Dosing schedule	Monthly IM injections	Single IM injection per season	Single IM injection per season

In recent years, novel mAbs have been developed. Nirsevimab became the first to gain approval in Europe in October 2022,^21^, ^22^ followed by US market authorization in June 2023,^23^ and a landmark WHO recommendation for global use in June 2025.^24^ Soon after, clesrovimab received FDA approval,^25^ expanding immunoprophylaxis options for neonates and infants. Thanks to molecular modifications that extend their half‐life, both mAbs provide full‐season protection with a single injection, enabling broad administration to all infants during their first RSV season.

The implementation of nirsevimab during the 2023–2024 epidemic season, particularly in France,^26^, ^27^, ^28^ Spain,^29^, ^30^ and parts of the United States,^31^ has provided the first robust real‐world data. These large‐scale immunization campaigns, often reaching 80% of eligible newborns, have demonstrated remarkable effectiveness, with a reduction in RSV‐related hospitalizations ranging from 70% to 90%,^32^ in line with findings from clinical studies.^17^, ^33^ Its favorable safety profile was also reaffirmed at scale, with no new pharmacovigilance concerns.^34^ In parallel, clesrovimab approval is supported by the recent results from clinical studies.^25^, ^35^, ^36^ In a phase 2b/3 trial, it showed 84.2% (95% CI, 66.6–92.6) reduction in RSV‐associated hospitalizations.^36^

Together, these long‐acting mAbs signal a paradigm shift, from targeted prophylaxis in high‐risk infants to a universal, population‐based strategy encompassing the entire birth cohort. The emergence of clesrovimab adds strategic value by fostering competition, promoting cost‐efficiency, and reinforcing the sustainability of universal RSV immunoprophylaxis.

Yet, clinical success is just one dimension of this evolving field. A deeper understanding of the molecular and immunological mechanisms that govern the interplay between virus, antibody, and host is critical to ensuring the long‐term viability of this approach and guiding the next generation of interventions. This narrative review explores these mechanisms, with particular focus on recent data from nirsevimab and clesrovimab. We will also review current insights into prophylactic failure, or “infectious breakthrough,” by addressing both viral factors (e.g., antigenic escape) and host factors (e.g., pharmacokinetics and immunogenicity). This analysis ultimately seeks to identify critical unanswered questions and existing knowledge gaps, in order to provide strategic guidance for the development of next generation prophylactic agents against RSV and other viral pathogens. Thus, a targeted literature search was conducted in PubMed, Google Scholar, and bioRxiv databases for articles published until August 2025, using combinations of the terms RSV, nirsevimab, clesrovimab, or monoclonal antibodies. Only peer‐reviewed studies in English were included.

## 2 MECHANISMS OF ACTION OF mAb

The efficacy of anti‐RSV mAbs is mediated by a dual mechanism that integrates the complementary functions of the antigen‐binding (Fab) and crystallizable (Fc) regions. The Fab fragment confers potent neutralizing activity through high‐affinity binding to conserved epitopes on the RSV fusion (F) protein, thereby directly blocking viral entry into host cells and preventing infection. In parallel, the Fc region of these human IgG1 kappa antibodies engages components of the host's innate immune system. By interacting with Fc gamma receptors (FcγRs) on effector cells and activating the complement cascade, the Fc domain initiates a range of effector functions, including antibody‐dependent cellular cytotoxicity (ADCC), phagocytosis (ADCP), and complement‐dependent cytolysis (CDC).^37^ Together, these actions promote the clearance of infected cells and help shape the broader antiviral immune response, underscoring the therapeutic potential of these antibodies beyond simple neutralization. While Fc‐mediated effector mechanisms are well established for conventional IgG1 antibodies, their contribution to the in vivo efficacy of extended–half‐life antibodies remains largely theoretical. These functions, though biologically plausible and demonstrated in vitro, have not yet been confirmed as determinants of clinical protection, as discussed below.

### 2.1 The dual action of neutralization and Fc‐mediated immune functions

#### 2.1.1 Neutralization

**FIGURE 2:**
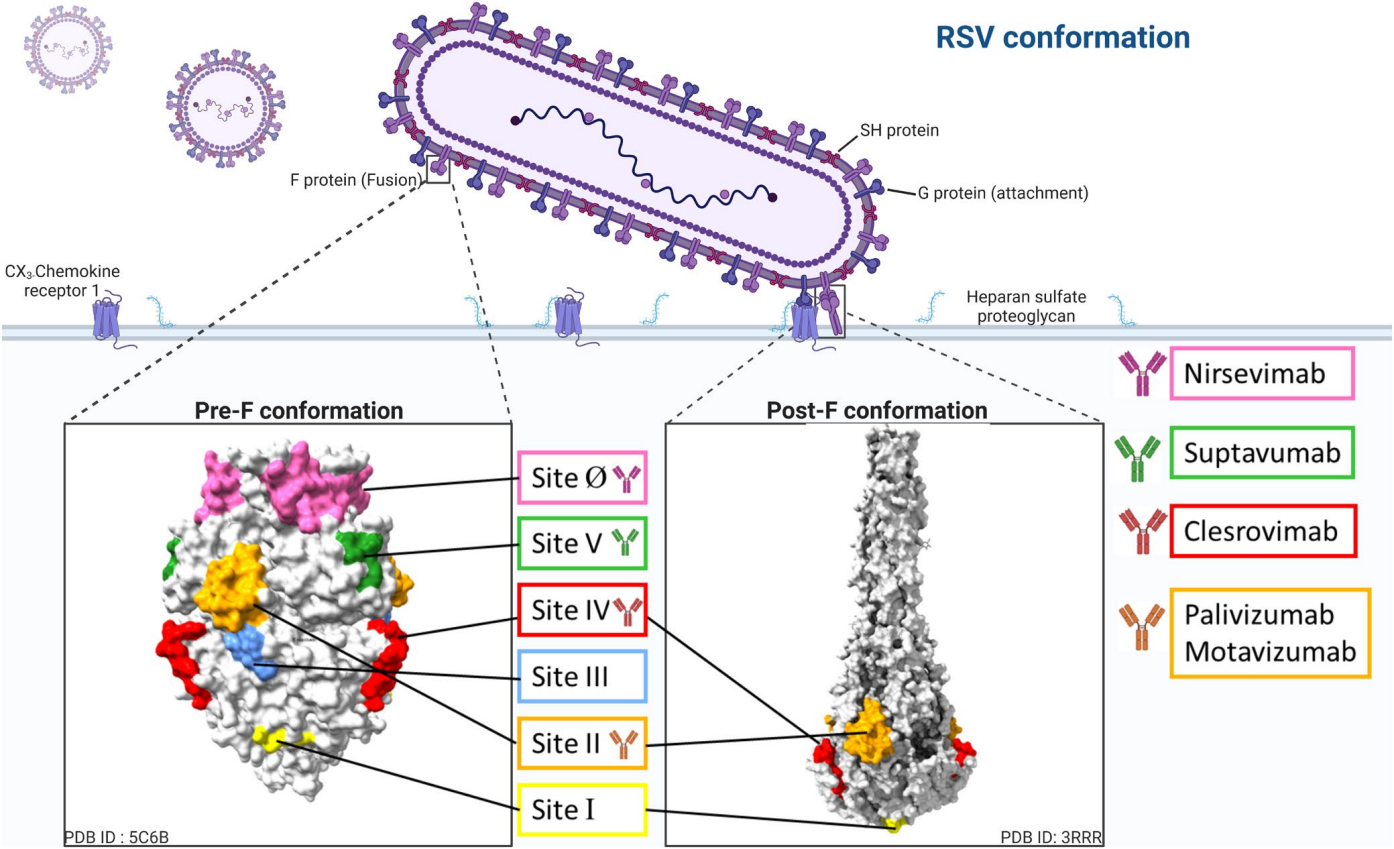
Structural mapping of antigenic sites on the RSV fusion (F) protein and monoclonal antibody targets. The pre‐fusion (pre‐F) and post‐fusion (post‐F) conformations expose distinct epitopes. Palivizumab binds site II, present in both states, whereas nirsevimab (site Ø) and clesrovimab (site IV) stabilize the pre‐F trimer, thereby preventing viral entry and conferring enhanced neutralization potency. SH: Small hydrophobic protein; pre‐F: Pre‐fusion conformation of F protein; post‐F: Post‐fusion conformation of F protein. Created in BioRender.

RSV is an enveloped, single‐stranded RNA virus. Its viral envelope contains glycoproteins essential for infection, notably the attachment protein (G) and the fusion protein (F) (Figure 2).^38^ The F protein is a class I glycoprotein required for fusion of the virus and host cell membranes, enabling virus entry and cell‐to‐cell propagation (syncytia formation).^38^ It is therefore the main target of preventive mAbs and vaccine development particularly as it is a highly conserved structure. The function of the F protein depends entirely on an irreversible conformational change, from a metastable pre‐fusion state (pre‐F) to a highly stable post‐fusion state (post‐F). The pre‐fusion form is the one present at the virion surface. Each conformation exposes several key antigenic sites (conserved between the two conformations or not) and are targets for neutralizing antibodies in pre‐F conformation^16^, ^39^ (Figure 2). This structural transition is therefore critical to understanding the mechanism of mAbs.

Palivizumab which has been developed (since 1996) before the detailed characterization of the pre‐F structure (Figure 1), targets a conformational epitope called site II.^13^, ^14^ This site is present on both the pre‐F and post‐F conformations. Although palivizumab is capable of inhibiting viral replication and fusion, its epitope is not associated with the same level of neutralizing power as sites exclusive to the pre‐F conformation, such as site Ø.^40^

Nirsevimab was developed (since 2022) following the recent discovery of the highly conserved Ø site specific to the pre‐F form.^16^ By binding simultaneously to the F1 and F2 subunits of the protein, nirsevimab acts as a molecular “clamp”, blocking the F protein in its pre‐fusion state and preventing viral entry into the host cell.^16^ Nirsevimab's enhanced neutralizing potency is largely attributed to its high‐affinity binding to the site Ø.

Clesrovimab (since 2023) targets the highly conserved site IV, a preferentially pre‐F site.^41^ By binding to this specific region with very high affinity, clesrovimab effectively stabilizes the pre‐F trimer, preventing it from undergoing the transition to its post‐fusion state.

Although both nirsevimab and clesrovimab lock the F protein in its pre‐F conformation, their distinct binding sites represent a remarkable example of molecular convergence, where different antibodies have evolved to neutralize the same viral machinery by targeting slightly different vulnerabilities on the pre‐F protein (Table 1). Direct comparative analyses further underscore the superiority of these engineered mAbs, as infants receiving nirsevimab show approximately 10‐fold higher RSV neutralizing titers than those administered monthly palivizumab injections.^40^

#### 2.1.2 Fc‐mediated immune response (Figure 3)

**FIGURE 3:**
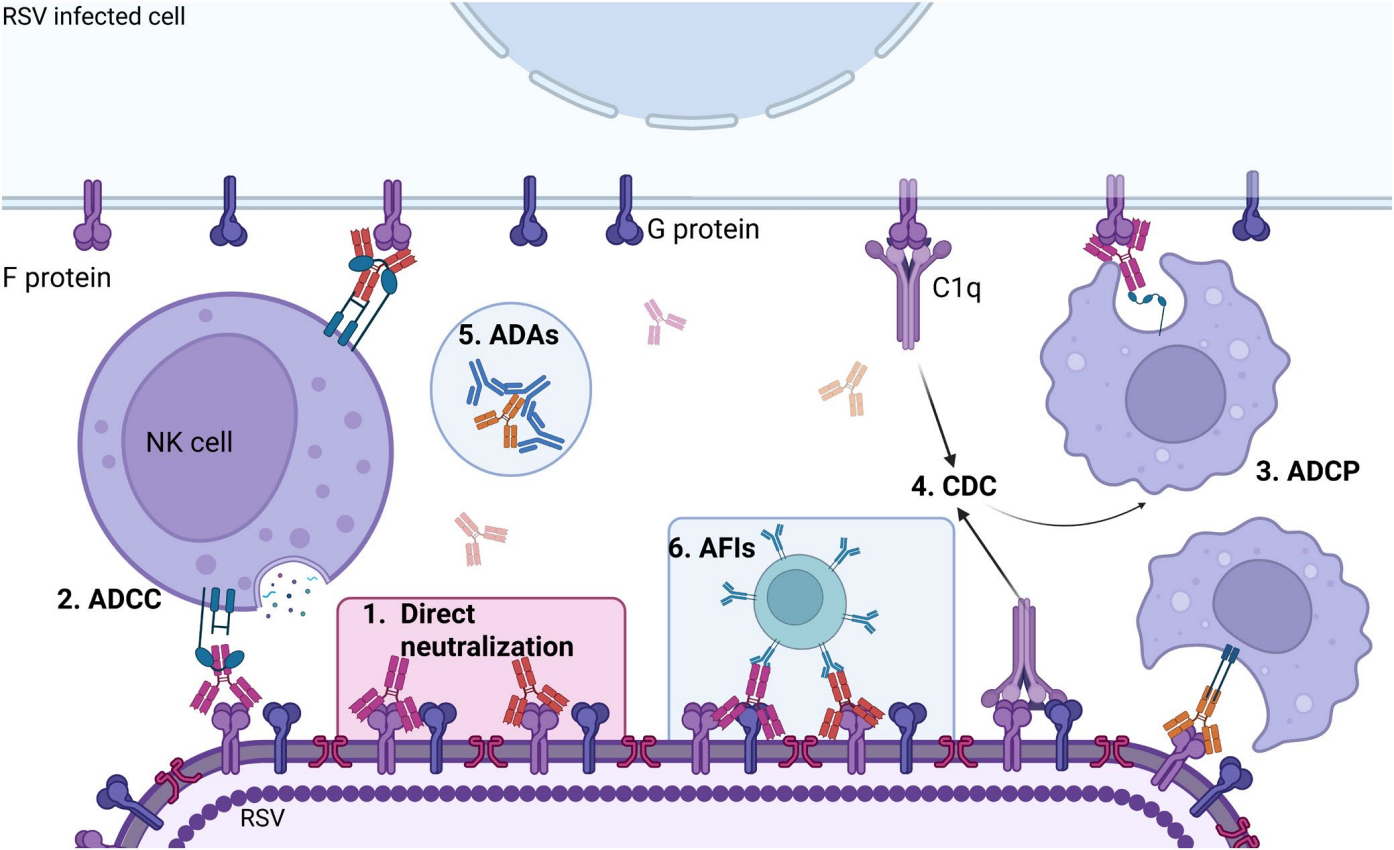
Mechanisms of action and immune modulation by RSV‐targeting monoclonal antibodies (mAbs). Monoclonal antibodies can neutralize RSV by directly binding to the F glycoproteins on the viral surface.^38^ In addition, Fc‐mediated effector functions contribute to viral clearance, including antibody‐dependent cellular cytotoxicity (ADCC) via NK cells,^42^ antibody‐dependent cellular phagocytosis (ADCP) by phagocytes,^43^ and complement‐dependent cytotoxicity (CDC) initiated by C1q binding.^44^, ^45^ Immune evasion can occur through the induction of anti‐drug antibodies (ADAs), which may neutralize the therapeutic mAb.^41^ Furthermore, antibody feedback inhibition (AFI) may impair B cell activation and antibody production by masking antigenic epitopes,^46^ potentially blunting long‐term humoral immunity. F protein: Fusion protein; G protein: attachment protein; RSV: Respiratory Syncytial Virus; NK cell: Natural Killer cell. Created in BioRender.

- Antibody‐Dependent Cellular Cytotoxicity (ADCC): This process is primarily mediated by Natural Killer (NK) cells. When an IgG1 antibody binds to viral antigens expressed on the surface of an infected cell, its Fc region is recognized by FcγRIIIA (CD16) receptors on NK cells.^42^ This cross‐linking activates the NK cell, causing it to release cytotoxic granules containing perforin and granzymes. Perforin creates pores in the target cell's membrane, allowing granzymes to enter and induce apoptosis, effectively destroying the infected cell and preventing further viral replication.^42^
- Antibody‐Dependent Cellular Phagocytosis (ADCP): This mechanism involves the engulfment and destruction of antibody‐coated infected cells by phagocytic cells, such as macrophages and neutrophils. Receptors on these phagocytes, particularly FcγRIIa on macrophages, bind to the Fc portion of the antibodies coating the target cell. This binding signals the phagocyte to internalize the infected cell into a phagosome, which then fuses with a lysosome to form a phagolysosome, where the cell and its viral contents are degraded by enzymes.^43^, ^47^
- Antibody‐Dependent Complement Deposition (ADCD) and Complement‐Dependent Cytotoxicity (CDC): When multiple IgG1 antibodies bind to antigens on a pathogen or an infected cell, their Fc regions can be recognized by C1q, the initial component of the classical complement pathway. This triggers a proteolytic cascade that leads to the deposition of complement proteins (ADCD) on the target surface, which can enhance phagocytosis. The cascade can culminate in the formation of the membrane attack complex, which inserts pores into the cell membrane, causing osmotic lysis and cell death (CDC).^44^, ^45^

Although palivizumab, nirsevimab and clesrovimab are all IgG1 mAbs and therefore inherently capable of engaging Fc‐mediated effector functions, the extent to which these mechanisms contribute to their overall activity varies depending on their specific F protein targets (Table 1).

For palivizumab, in vitro studies have shown that it mediates ADCC via FcγRIIIa and ADCP via FcγRIIa. It is also capable of activating the classical complement pathway.^48^ However, the in vivo relevance of these Fc‐mediated effector functions remains unclear, and neutralization is generally considered its primary mode of action, as definitive evidence from preclinical models is lacking.^49^ In vitro nirsevimab assays showed that it elicits ADCP and ADCD activities comparable to palivizumab, albeit with reduced ADCC potential. Importantly, preclinical studies in cotton rats using an Fc‐inactivated variant of its precursor (MEDI8897‐TM) demonstrated equivalent protection against RSV challenge compared to the intact antibody, confirming that direct neutralization is both the primary and sufficient mechanism underlying its in vivo efficacy.^49^ For clesrovimab, its ability to engage Fc‐mediated effector functions is presumed, given its IgG1 structure. However, its in vivo reliance on Fc‐mediated effector functions has not yet been delineated in published studies, highlighting a key gap in our understanding. This comparative landscape underscores a strategic evolution in mAb design, exemplified by nirsevimab, where optimizing neutralization potency and extending half‐life have taken precedence over enhancing Fc effector activity.

### 2.2 YTE engineering of nirsevimab: a key to single‐dose seasonal protection

The key pharmacological innovation of the new mAbs lies in Fc region engineering, specifically the introduction of the ‘YTE’ modification, a triple amino acid substitution (M252Y/S254T/T256E) designed to extend half‐life.^50^ This modification significantly enhances the antibody's affinity for the neonatal Fc receptor (FcRn), which binds IgG in acidic endosomes, prevents lysosomal degradation, and recycles it back to the cell surface for release at neutral pH.^51^ By strengthening this interaction, the YTE modification significantly extends antibody persistence in vivo. Nirsevimab serum half‐life in infants is approximately 71 days, over three times longer than palivizumab, which has half‐life of 18–21 days.^40^ Early data for clesrovimab indicate an apparent half‐life of about 45 days.^35^ Consequently, a single intramuscular dose of nirsevimab sustains exceptionally high neutralizing antibody (nAb) titers throughout the 5‐month RSV season, with >140‐fold increases over baseline at 1 month post‐dose and >50‐fold elevations after 5 months. Notably, titers remain over sevenfold above baseline at 1 year, indicating prolonged protection.^40^ Similarly, clesrovimab induces dose‐dependent neutralizing responses persisting for several months, with 16‐ to 23‐fold rises above baseline after 5 months in first trials.^35^

## 3 ANALYSIS OF THERAPEUTIC FAILURES: BREAKTHROUGH INFECTIONS

Despite their high overall efficacy, palivizumab, nirsevimab and clesrovimab do not provide complete protection, as a small proportion of immunized infants still develop RSV LRTI in their first months of life.^32^ Elucidating the mechanisms underlying these breakthrough infections is key to delineating the limitations of current prophylactic strategies and guiding the development of more effective interventions. These failures can be broadly classified into two categories: virus‐related factors and host‐related factors.

### 3.1 Virus‐Dependent Factors: Antigenic Escape

RSV, like other RNA viruses, relies on an error‐prone RNA polymerase that generates extensive genetic diversity within viral populations. When a highly specific mAb is widely administered, strong selective pressure can promote the emergence and spread of viral variants harboring mutations in the antibody's target epitope, facilitating antigenic escape.^52^, ^53^ The phenomenon of mAb resistance was first documented with palivizumab. Although resistance mutations are rare, some have been shown to confer near‐complete resistance to neutralization but with a reduced replicative fitness compared to wild‐type virus, limiting their ability to persist.^54^, ^55^, ^56^, ^57^ Thus, no palivizumab escape mutants have yet achieved sustainable circulation. In contrast, the recent failure of suptavumab, a mAb targeting antigenic site V of the RSV F protein (Figure 2), highlights the risks of viral escape on a larger scale. The suptavumab phase III trial failure was driven by the emergence and widespread circulation of an RSV‐B strain harboring mutations within the targeted epitope, which rendered the antibody ineffective (Figure 1).^58^ However, the resistance observed in the suptavumab trial occurred independently of the selective pressure induced by the antibodies. The widespread introduction of nirsevimab has heightened the critical need for vigilant viral escape surveillance. An analysis of 5675 RSV‐A and RSV‐B sequences spanning 1956–2021 revealed that fewer than 1% harbored mutations within the nirsevimab binding site, while no mutations were found in the clesrovimab binding site, indicating remarkable epitope stability over decades.^52^, ^58^ However, genomic surveillance from 2013 to 2023, prior to large‐scale use of these mAbs, identified three pre‐existing mutations in site Ø that became predominant in RSV‐B strains despite no selective antibody pressure.^52^ These mutations, which have no neutralizing power, are still widespread in recent clinical samples, indicating the possible acquisition of stable mutations. In contrast, no mutations emerged in the clesrovimab‐targeted site IV. Real‐world genomic studies conducted during the 2023–2024 RSV season in France,^59^ Australia^60^ or the USA^61^ analyzed hundreds of breakthrough infection sequences, finding that over 99% retained sensitivity to nirsevimab (rare mutations in RSV‐B).

This scenario parallels other viral infections, notably SARS‐CoV‐2, where rapid evolution of the Spike protein, especially within the receptor‐binding domain, led to multiple variants of concern such as Omicron sub‐lineages BA.1, BA.2, and BA.4/BA.5.^62^ These variants accumulated mutations that abrogated the efficacy of first‐generation mAbs.^63^ However, as with the suptavumab case, no causal link has been established between the prophylactic or therapeutic use of anti‐SARS‐CoV‐2 mAbs and the emergence of resistant mutants. The analogy should therefore be interpreted with caution. RSV exhibits a markedly slower molecular clock and limited antigenic drift compared to coronaviruses limiting the risk of emerging mutants, with stable F‐protein epitopes persisting over decades. Together, these insights emphasize the dynamic interplay between viral evolution and antibody‐mediated interventions, reinforcing the need for continuous surveillance and adaptive strategies in RSV prophylaxis.

### 3.2 Host‐dependent factors: variability in response and exposure

Viral escape is not the sole cause of breakthrough infections; host‐specific factors, particularly individual characteristics of the immunized infant, can also contribute to prophylaxis failure. First, recent real‐world data from Spain described the clinical characteristics of 69 infants hospitalized for RSV LRTI of whom 65% (45 patients) were breakthrough cases.^64^ The clinical severity was comparable between breakthrough and non‐breakthrough cases, suggesting that no clinical marker currently distinguishes these groups and that host‐related factors may contribute to breakthrough events.

The pharmacokinetics of mAbs in infants differ markedly from adults and cannot be simply extrapolated.^65^, ^66^ Specific physiological characteristics of early life contribute to this complexity and notably: infants have a higher proportion of total body water, expanding the volume of distribution for hydrophilic macromolecules like mAbs^65^; they exhibit enhanced tissue perfusion and more permeable capillary networks, facilitating greater extravasation of large molecules^65^, ^66^; additionally, accelerated protein turnover combined with physiologically low endogenous IgG levels can alter FcRn recycling efficiency, leading to increased mAb clearance.^67^ Together, these factors result in considerable interindividual variability in mAb exposure, with some infants displaying serum concentrations below protective thresholds, thereby elevating their risk of breakthrough infections.^68^, ^69^

Additionally, the host immune system can recognize therapeutic mAbs as foreign proteins and mount an immune response by producing anti‐drug antibodies (ADAs) (Figure 3). These ADAs may neutralize directly mAb's activity or create immune complexes that accelerate antibody clearance, thereby reducing half‐life and therapeutic efficacy. In the pivotal IMpact‐RSV palivizumab trial, only 0.7% of infants developed ADAs after the fourth monthly dose.^13^ However, pharmacokinetic analysis revealed that children with high ADA titers experienced approximately a 20% increase in palivizumab clearance, potentially lowering drug exposure.^70^ While rare hypersensitivity reactions have been reported, their association with ADAs remains unclear. Nirsevimab shows a higher ADA incidence, with 6.1% of infants developing ADAs post‐injection in the MELODY trial, compared to 1.1% in placebo recipients.^17^ These ADAs primarily target the YTE‐modified Fc region^34^, ^41^; although ADA‐positive infants exhibited reduced nirsevimab serum concentrations late in the RSV season, no adverse effects on safety or overall efficacy were observed during the critical protection window. Clesrovimab, which also displays the YTE modification, demonstrated even higher ADA rates, approximately 23% over 545 days of follow‐up in a phase 1b/2a trial.^35^, ^41^ Most high‐titer ADAs emerged after day 150 and correlated with RSV exposure. These ADAs predominantly targeting the YTE Fc region, showed minimal reactivity against the Fab domain, and did not impact clesrovimab's pharmacokinetics or safety profile (Figure 3).

## 4 IMMUNOLOGICAL CONSEQUENCES OF PASSIVE IMMUNIZATION WITH MONOCLONAL ANTIBODIES

The administration of a highly potent exogenous antibody to an infant whose immune system is still maturing, raises important questions regarding potential long‐term immunological consequences. Key concerns include whether passive immunization might interfere with the natural acquisition of immunity by blunting antigen exposure, the theoretical risk of antibody‐dependent enhancement (ADE) of disease upon subsequent RSV encounters, and of antibody feedback inhibition. These concerns are especially relevant in early life, when immune imprinting can shape future responses.

### 4.1 Impact on natural immunity development and post‐exposure responses: coexistence of passive protection without delaying disease burden to year two

A major concern surrounding passive immunization is the potential interference with the development of natural, long‐lasting immunity, specifically, the idea that exogenously administered mAbs might neutralize RSV before the infant's immune system can recognize it, thereby preventing the generation of immunological memory. However, clinical trial data from both palivizumab and nirsevimab strongly counter this hypothesis.^40^, ^71^ Serological analysis from the MELODY trial demonstrated that infants who received nirsevimab and were subsequently exposed to RSV developed endogenous antibody responses, particularly against the post‐F form, at rates comparable to infected infants in the placebo group.^40^ This indicates that RSV exposure still occurred in the presence of passive protection, enabling the maturation of adaptive immunity. Similar outcomes were reported in head‐to‐head studies comparing palivizumab and nirsevimab, where both prophylaxis groups showed evidence of natural immune priming upon RSV exposure, likely due to subclinical or mild infections.^72^ This coexistence of immediate passive protection with the opportunity for immune imprinting represents an optimal immunological profile for RSV prophylaxis in infancy. In addition to preventing acute severe disease, early RSV prophylaxis may confer broader health advantages that extend beyond virological protection. By attenuating viral replication and airway inflammation, it could probably reduce the subsequent risk of recurrent wheezing and early‐childhood asthma, as suggested by epidemiologic links between severe RSV infection and asthma.^5^, ^6^ Moreover, recent studies have reported that the introduction of nirsevimab led to a 23.7% reduction (95% CI −37.6 to −9.7) of acute otitis rate in infants aged <12 months.^73^ Crucially, concerns about a delayed disease burden shifting to the second year of life have not been supported by long‐term follow‐up data. Decades of real‐world use of palivizumab have shown no increase in severe RSV cases during the second year among previously protected infants; instead, a persistent reduction in RSV‐related illness has been observed.^74^ Similarly, infants who received nirsevimab in the MELODY trial and were monitored through the subsequent RSV season showed no increase in medically attended RSV‐LRTI compared to the placebo group.^75^ These findings have been recently corroborated by real‐world surveillance studies which reported no increase in severe RSV disease among children entering their second season.^76^, ^77^ Lack of rebound disease indicates that early‐life prophylaxis does not hinder the development of natural immunity, but rather contributes to a sustained decrease in RSV susceptibility. Collectively, these findings demonstrate that passive immunization with mAbs like nirsevimab offers both immediate and durable benefits, preventing early severe disease while still permitting the development of protective natural immunity.

### 4.2 Theoretical risk of ADE: lessons from the formalin‐inactivated vaccine and absence of evidence with palivizumab, nirsevimab, and clesrovimab

The history of RSV prevention is indelibly shaped by the failure of the formalin‐inactivated vaccine in the 1960s, which led to severe enhanced respiratory disease in vaccinated infants upon natural RSV exposure, with hospitalization rates approaching 80% and two recorded deaths.^9^ Subsequent analyses identified a maladaptive immune response characterized by Th2‐skewed cytokine profiles, pulmonary eosinophilia, and the generation of low‐avidity, non‐neutralizing antibodies that formed immune complexes in the lungs.^78^, ^79^ This event firmly established ADE as a critical theoretical risk for any RSV immunization strategy. However, decades of clinical experience with palivizumab and extensive trial and real‐world data for nirsevimab and clesrovimab have provided no evidence of ADE. Importantly, follow‐up through the second RSV season, when circulating antibody levels are lowest and potentially sub‐neutralizing, has shown no increase in disease severity among immunized infants.^75^, ^80^

This absence of ADE reflects the fundamental difference of the mechanistic approach as mAbs are highly specific and potently neutralizing, blocking viral entry at the earliest step and thereby suppressing replication and limiting antigen load. By preventing the conditions that promote immune complex deposition and inflammatory amplification, these mAbs effectively circumvent the pathogenic pathways associated with ADE. Their safety profile underscores a modern immunological principle: the quality and specificity of the antibody response are more critical for protection than sheer antibody quantity.

### 4.3 Antibody feedback inhibition: an avenue for exploration

Beyond ADE, another nuanced immunological mechanism warrants consideration: antibody feedback inhibition (AFI). This phenomenon occurs when high concentrations of specific antibodies against a given epitope modulate the subsequent B‐cell response upon antigen re‐exposure.^46^ In certain contexts, this mechanism may be beneficial; by masking the immunodominant epitope, pre‐existing antibodies can redirect the immune system toward subdominant regions of the antigen, thereby promoting a broader and more diverse antibody repertoire. Conversely, this feedback can dampen the overall B‐cell response, potentially reducing the magnitude of antibody production. Evidence from SARS‐CoV‐2 studies has shown that individuals who received mAb therapy prior to vaccination mounted a more diversified, yet quantitatively lower, antibody response.^46^, ^81^, ^82^ In the context of RSV prophylaxis, this raises a critical and still unresolved research question: does the potent and epitope‐specific action of nirsevimab and clesrovimab, particularly when targeting the highly conserved sites Ø and IV respectively, influence the quality or breadth of the infant's endogenous B‐cell response during natural RSV infection? If such diversification occurs, it could enhance long‐term immune resilience, providing broader protection against future viral variants. Understanding how passive immunization with a highly potent, epitope‐focused mAb shapes the adaptive immune landscape, particularly the diversity, affinity maturation, and epitope targeting of endogenous B‐cell responses, as well as the quality of T‐cell activation, could reveal fundamental insights into how early‐life immune systems adapt to viral exposure in the presence of passive immunity.

Maternal immunity can shape early‐life immune development through immune imprinting,^83^, ^84^, ^85^ but whether RSV establishes such imprinting remains unclear. Influenza induces durable, cross‐reactive CD4^+^ and CD8^+^ T‐cell memory to conserved internal proteins, enabling strong recall responses and reduced disease severity later in life.^86^, ^87^ In contrast, RSV elicits weaker, short‐lived cellular memory due to infant immune immaturity, viral suppression of antiviral pathways, and limited boosting from reinfections, often resulting in Th2/Th17‐skewed responses.^88^, ^89^ Recent profiling by Nziza et al. identified age‐dependent RSV humoral signatures and a window of early‐life vulnerability but found no evidence that maternal RSV antibodies imprint or alter infant RSV humoral immunity.^90^ Although maternal antibodies can impair vaccine responses, as shown for measles,^91^ their influence on post‐infection RSV immune maturation remains unresolved. Such research and knowledge hold the potential to inform not only RSV prevention strategies but also broader principles of vaccine design and immunological development in infancy.

Taken together, these findings demonstrate that mAbs provide robust and durable protection without compromising the natural development of immunity. However, as these antibodies redefine early viral exposure, they may also modulate the architecture of early immune responses. Further research is therefore needed to elucidate how passive neutralization in infancy influences the maturation and long‐term imprinting of the adaptive immune system. A comprehensive understanding of these mechanisms will be essential to balance the clear short‐term benefits of protection with potential long‐term effects on immune education, ensuring that mAbs ultimately strengthen, rather than attenuate, lifelong antiviral competence.

## 5 UNRESOLVED QUESTIONS AND FUTURE STRATEGIES FOR RSV PROPHYLAXIS

The success of nirsevimab has marked a turning point in RSV prevention, demonstrating that a single‐dose mAb can provide effective, season‐long protection in infants. However, this achievement also reframes the research agenda, raising critical questions about long‐term immunity, viral evolution under selective pressure, and population‐level impact as developed above. To ensure the durability of this success and to guide the development of next‐generation interventions, it is essential to identify current knowledge gaps, and to explore innovative strategies. This evolving landscape calls for a dynamic integration of clinical, immunological, and genomic surveillance to adapt RSV prevention to future challenges.

### 5.1 Closing critical gaps: long‐term RSV evolution, antibody protection, and cellular immunity under passive immunization

The widespread use of nirsevimab could exert substantial selective pressure on the RSV Ø epitope, potentially leading to the development of resistant viral strains. However, it is difficult to assess the magnitude of this pressure. Although infants experience the most severe clinical RSV infections, they account for an unknown proportion of total cases. Recent epidemiological data indicate that almost 50% of individuals become infected each year, accounting for the majority of RSV transmission.^92^ This complicates efforts to model the drug's selective impact across age groups. Moreover, the fitness of resistant variants—a critical determinant of their spread—is currently undefined. If resistance mutations impose a fitness cost, their transmission may be self‐limiting. Conversely, fitness‐neutral or advantageous mutations could facilitate rapid dissemination. Without empirical data on these trade‐offs, predictions remain speculative. The development of additional mAbs, such as clesrovimab, offers a potential strategy to reduce selective pressure by targeting multiple viral epitopes simultaneously. Deploying a combination of mAbs with complementary epitope specificities could therefore slow the emergence of escape variants. Continuous, global molecular surveillance of circulating RSV strains is still essential to promptly detect variant expansions and guide adaptive prophylaxis strategies.

At the population level, high mAb titers correlate with strong protection; however, the antibody concentration threshold required to confer protection remains undefined. Establishing a reliable serological correlate of protection would improve the understanding of breakthrough infections, help identify infants at risk despite prophylaxis, and optimize dosing regimens for future mAbs, particularly among highly vulnerable groups.

Although nirsevimab does not significantly interfere with the development of endogenous antibody responses following natural RSV exposure,^40^, ^72^ its influence on T‐cell memory formation is much less characterized. Key questions remain about whether the reduced viral load during breakthrough infections affects the magnitude, quality, or durability of CD4^+^ and CD8^+^ T‐cell memory.^93^, ^94^ This is of critical importance since cellular immunity plays a vital role in viral clearance and protection against severe reinfections. Long‐term immunological studies focusing on cellular responses are therefore necessary to address these gaps.^94^ Additionally, monitoring changes in the B‐cell response is crucial to determine if early passive immunization with mAbs induces long‐term effects such as immune blunting, as it has been observed in some vaccine studies.^95^, ^96^

### 5.2 The next generation of prophylactic antibodies

#### 5.2.1 Bispecific antibodies: dual‐epitope targeting to overcome viral escape

Building on breakthroughs in oncology and HIV research, bispecific antibodies (bsAbs) offer a highly promising approach for RSV prevention.^97^, ^98^ An anti‐RSV bsAb can be engineered to simultaneously target two distinct, non‐overlapping epitopes on the F protein, such as site Ø along with another conserved region, thereby significantly increasing the genetic barrier to viral escape. This dual targeting means the virus should acquire mutations in both sites to evade neutralization, which is an unlikely event. More innovative strategies are also being explored, including anti‐idiotypic bsAbs that do not neutralize the virus directly but instead activate specific B‐cell populations pre‐programmed to produce neutralizing antibodies, effectively functioning as a precision vaccine.^99^

#### 5.2.2 Nanobodies: unlocking inhaled delivery and enhanced tissue penetration

Nanobodies single‐domain antibody fragments derived from camelid heavy‐chain‐only antibodies, offer a compact (~12–15 kDa), highly stable scaffold that is cost‐effective and easy to produce. Their small size and exceptional thermostability make them ideal therapeutic candidates, with the added advantage of potential nebulized administration for direct delivery to the respiratory tract.^100^ For RSV, nanobodies targeting the pre‐F protein have demonstrated potent neutralizing activity in vitro and protective efficacy in mouse models, with biophysical properties well‐suited for aerosol formulation.^101^

Moreover, their reduced size allows them to access cryptic or recessed epitopes on the F protein that conventional mAbs may not reach, potentially enhancing neutralizing breadth and lowering the risk of escape mutations.^102^ This technology thus represents a promising new avenue for localized, rapid‐acting RSV therapies that can complement existing systemic prophylaxis strategies.

#### 5.2.3 Artificial intelligence for development of new products

Artificial intelligence (AI) is reshaping drug and therapeutic antibody development by accelerating discovery, enhancing precision, and lowering costs. Through epitope prediction, structural modeling, and de novo protein design, AI facilitates the identification of immunogenic targets and the engineering of stabilized antigens. For instance, computational scaffolding has enabled RSV immunogens design focused on neutralizing epitopes,^103^ while high‐throughput in silico screening and generative AI have optimized antigen stability and immunogenicity, leading to vaccine candidates with enhanced potency,^104^ and produced synthetic nanobodies targeting RSV F protein.^105^ These advances show how AI‐driven approaches are set to redefine the entire landscape of drug development and rapidly revolutionize treatments for numerous infections that have long resisted new therapeutic breakthroughs.

### 5.3 Recommendations for future research and development

A comprehensive evaluation of the achievements, limitations, and future potential of mAbs against RSV highlights several key strategic priorities:

1. Strengthen integrated surveillance: Sustained and expanded surveillance efforts are critical, combining high‐resolution genomic monitoring to detect emerging resistance mutations with longitudinal real‐world studies to characterize immunological assessments, long‐term safety and durability of protection, in pediatric cohorts. This dual approach will enable real‐time evaluation of viral evolution and the durability and quality of immune protection conferred by prophylaxis. Moreover, particular emphasis should be placed on populations in low‐ and middle‐income countries, where RSV burden is highest and where pharmacokinetic profiles, exposure dynamics, and comorbidity patterns may differ substantially from those in high‐income settings.
2. Define correlates of protection: Rigorous prospective studies are needed to define antibody concentration thresholds that correlate with clinical protection at the individual level. Such serological benchmarks are vital for tailoring dosing regimens, optimizing prophylactic efficacy, and informing regulatory and clinical decision‐making.
3. Deepen understanding of breakthrough infections: breakthrough cases following nirsevimab prophylaxis are uncommon, but anticipating and understanding their virological, immunological, and clinical characteristics remain essential. Distinguishing true prophylactic failure from expected partial protection or variable host responses will inform refinement of prevention strategies and targeted surveillance.
4. Advance next‐generation therapeutics: Priority should be given to developing bispecific antibodies targeting multiple RSV epitopes to substantially raise the barrier to viral escape. Concurrently, exploring the therapeutic potential of nanobodies, particularly for inhaled delivery, could address the current unmet need for rapid‐acting treatments in breakthrough infections or unprotected populations.

## 6 CONCLUSION

The advent of prophylactic mAbs, most notably nirsevimab and recently clesrovimab, has initiated a transformative era in the prevention of severe RSV infections in infants. Yet, this success must not obscure the remaining challenges. Breakthrough infections, though uncommon, highlight the multifactorial nature of prophylactic failure, arising from both viral and host‐related dynamics.

As the field moves forward, RSV prophylaxis stands at a critical juncture. Open questions regarding the virus's evolutionary response to population‐wide mAb pressure, the definition of protective antibody thresholds at the individual level, and the implications for cellular immunity remain to be addressed. Innovative next‐generation platforms, such as bsAbs designed to mitigate resistance and nanobodies optimized for inhaled, offer exciting avenues for therapeutic expansion. By applying insights gained from current achievements and limitations, the field is now positioned to consolidate and extend the foundational success of nirsevimab, with the ultimate goal of sustainably reducing the global burden of RSV in early childhood.

## AUTHOR CONTRIBUTIONS

**Etienne Bizot:** Conceptualization; methodology; writing – original draft. **Vincent Portet Sulla:** Writing – review and editing. **Christelle Vauloup‐Fellous:** Writing – review and editing. **Marie‐Anne Rameix‐Welti:** Writing – review and editing. **Vincent Gajdos:** Writing – review and editing. **Nabila Seddiki:** Conceptualization; methodology; writing – review and editing; supervision.

## CONFLICT OF INTEREST STATEMENT

No conflict of interest.
